# The emergence of identity, agency and consciousness from the temporal dynamics of neural elaboration

**DOI:** 10.3389/fnetp.2024.1292388

**Published:** 2024-04-02

**Authors:** Riccardo Fesce

**Affiliations:** Department of Biomedical Sciences, Humanitas University, Medical School, Pieve Emanuele, Italy

**Keywords:** consciousness, identity, agency, emergent properties, network physiology, time elaboration

## Abstract

Identity—differentiating self from external reality—and agency—being the author of one’s acts—are generally considered intrinsic properties of awareness and looked at as mental constructs generated by consciousness. Here a different view is proposed. All physiological systems display complex time-dependent regulations to adapt or anticipate external changes. To interact with rapid changes, an animal needs a nervous system capable of modelling and predicting (not simply representing) it. Different algorithms must be employed to predict the momentary location of an object based on sensory information (received with a delay), or to design in advance and direct the trajectory of movement. Thus, the temporal dynamics of external events and action must be handled in differential ways, thereby generating the distinction between self and non-self (“identity”) as an intrinsic computational construct in neuronal elaboration. Handling time is not what neurons are designed for. Neuronal circuits are based on parallel processing: each bit of information diverges on many neurons, each of which combines it with many other data. Spike firing reports the likelihood that the specific pattern the neuron is designed to respond to is present in the incoming data. This organization seems designed to process synchronous datasets. However, since neural networks can introduce delays in processing, time sequences can be transformed into simultaneous patterns and analysed as such. This way predictive algorithms can be implemented, and continually improved through neuronal plasticity. To successfully interact with the external reality, the nervous system must model and predict, but also differentially handle perceptual functions or motor activity, by putting in register information that becomes available at different time moments. Also, to learn through positive/negative reinforcement, modelling must establish a causal relation between motor control and its consequences: the contrast between phase lag in perception and phase lead (and control) in motor programming produces the emergence of identity (discerning self from surrounding) and agency (control on actions) as necessary computational constructs to model reality. This does not require any form of awareness. In a brain, capable of producing awareness, these constructs may evolve from mere computational requirements into mental (conscious) constructs.

## 1 Introduction

Animals are bound to interact with the external reality, to fetch a prey or escape a predator, and to modify their pattern of movement and their internal integrated regulations in response to, or in prediction of, external changes and challenges. The temporal dynamics of external changes are comparable to, and sometimes more rapid than, the timings of internal adaptations and behavioural responses, and this must be taken into consideration in controlling the complex physiological networks and responses. For example, starting to run would produce a significant rise in plasma CO_2_ before the carotid and aortic glomi, and the central pH receptors, trigger an increase in ventilation; still, we do not feel shortness of breath when we start running, whereas we feel it when we stop! That is because the respiratory centre—and the cardiovascular control centre—are activated even before the exercise starts, and CO_2_ levels decrease initially during the exercise, while they do rise momentarily when the exercise is interrupted. Behavioural interactions with rapid external events may be even more problematic: a fly typically flies at about 7 km/h, i.e. 2 m/s; the time needed for the visual input to reach the human cortex, and the motor command to reach the muscles of the arm, is in the order of several tens of ms, neglecting any (significant) time for the computation of the appropriate response; in the meanwhile the fly will have travelled several centimetres, and possibly have changed its direction. So, “mission: almost impossible” for a human to catch a flying fly; possibly not for a lizard, given the much shorter distances nerve impulses must travel.

In all cases, appropriate responses and adaptations—either reactive or anticipatory—require that the delay in the body networks—both systemic and neural—be taken into consideration.

I argue here that, independent of how refined are the elaborative capabilities of a brain, two computational constructs must contribute to model reality to a level adequate to correctly interact with it: the first consists in the differentiation of the self from the external reality in such model, because the animal needs to treat differently the temporal dynamics of reality (perceived through sensory inputs with a delay), and of its own behaviour (that must be programmed and commanded in advance); the second is strictly correlated and consists in the resulting differentiation between what occurs out there and what is caused by the animal’s behaviour. There is no better definition for these two computational constructs than “identity” and “agency,” respectively. Whether meta-elaboration leads to a perception of such constructs, and transformation of identity and agency into pillars of self-consciousness, depends on the complexity of the brain of each animal; in all cases, both constructs do precede consciousness and are not generated by it.

## 2 Consciousness: representing or modelling reality?

A philosophical question, brought to centerstage by AI, is why we are so reluctant to attribute identity, agency, and consciousness to a machine, whereas we feel that animals, possibly even insects, must have some form of identity, and the capability of producing some kind of “experience,” of perception of “being there.” This property is generally considered as a product of consciousness, which in turn is generally perceived as the capacity to internally represent reality (external reality as well as the self) and adding to such representation the flavours of identity and agency. However, this is misleading because the general organization of the nervous system is not consistent with the purpose of ‘representing’ or ‘depicting’ reality.

The circuitry of the nervous system is based on the interaction between two general principles—divergence and convergence (Man et al., 2013)—that results in parallel processing of information. Each signal is typically sent to many neurons (divergence), and at each of them it is integrated with many other inputs (convergence). This has long been known: for example, in the frog retina “a ganglion cell is potentially related to [...] thousands of receptors. Conversely, [...], any receptor [...] must be related to hundreds of ganglion cells. Thus, many ganglion cells of different morphological types are looking at the same point of the visual field and through the same receptors” (Maturana et al., 1960). This leads to a “transformation of the visual image from a matrix of discrete point measurements of light intensities into a matrix of overlapping contexts” (ibid.).

So, no neuron examines a single bit of data, but all neurons elaborate relations among data, and patterns (e.g., Hubel and Wiesel, 1968). This way the sensory data stream is distributed into a myriad of paths and simultaneously recombined in innumerable ways, so that many possible readings can be compared and chosen among, to formulate the most likely interpretation of the incoming information. This will be the one reading that appears to be maximally consistent with the multiple, sometime conflicting, interpretations that the various computational modules involved are suggesting. In synthesis, this will not be an attempt at depicting reality but rather a ‘model’, an abstract — possibly consistent — scheme to interpret it.

The approach is particularly effective in analysing large sets of data that arrive simultaneously, such as, for example, visual information. A neuron receives many time-varying synaptic inputs, and at each moment their summation may or may not reach the threshold to generate an action potential. Thus, the output of the neuron constitutes a time-varying monitor of the possible presence, in the incoming data, of a specific pattern—the pattern it is designed to recognize. Since the information reaching the brain is continuously changing, for a network to be able to identify a specific pattern in the incoming data stream each neuron must be able to accurately select and isolate inputs that arrive truly synchronously. Neural networks can make this possible by restricting, thanks to inhibitory interneurons, the temporal window for synaptic input summation (see, e.g., Tremblay et al., 2016, and farther down, §2).

In addition to spatial patterns (relations among data from a simultaneously acquired set), neural networks must elaborate temporal patterns: this is obtained through two main strategies. On the one hand, neuronal networks can exploit the delay introduced by each synaptic connection (about 1 ms) to make it so that a set of inputs, processed in parallel by paths of different length (number of synapses), reach the same neuron with different, predetermined delays. This way, a temporal sequence is translated into a synchronous pattern, which can be specifically identified by the neuron; the delays introduced by neural paths can also undergo adaptation (Eurich et al., 2000). This method can be further refined by taking into consideration the attenuation and time delay suffered by synaptic signals that occur at dendritic sites removed from the cell body. The second strategy is used to analyse particularly rich temporal sequences, such as sound: exploiting the frequency-tuned responses of a battery of receptor cells in the cochlea, the rapidly time-varying pressure is converted in real time into a time-varying pattern of population spike firing — a tonotopic map — that represents the momentary (Fourier) transform of the sound into the frequency domain (Reimann, 2011): a wide band signal (20 kHz for humans, even higher frequencies for other animals) that largely exceeds the possibility of being translated into a modulated spike frequencies (a maximal rate of ∼300 spikes/s cannot reasonably reproduce frequencies higher than some tens of Hertz in a signal) is transformed into a “movie” made of a slower temporal sequence of sound ‘frames’ (synchronous patterns).

Handling time-varying information is crucial for the survival of every organism. However, producing a response in the brain may require several tens of ms (depending on the location in the body that receives the stimulus), and when intense processing is needed to extract significant information, neural circuits may take several hundred ms to perform complex feature extraction and attribution of meaning; similarly, programming and executing motor behaviours require at least tens of ms. This may not seem particularly relevant, as in normal life one usually does not care about being late by possibly half a second. But if one sees how a lizard manages to catch a flying insect, it becomes clear that a huge problem for the nervous system is to be able to compute and compensate for the delay that separates sensing from acting (a fly typically flies at up to 5 mph, i.e., ∼2 m/s, which means that a delay of 10 ms will make the lizard miss the fly by 2 cm, and never catch it).

Independent of the complexity of the brain, an animal that can detect an object (e.g., a prey) and move to get in contact with it needs to be able to ‘model’ what is happening: it does not have information to reproduce what is actually occurring in this moment, due (1) to the delay in receiving and elaborating sensory information, and (2) to the fact that any movement it might want to do must have been programmed and commanded in advance, and will only be sensed and reported by proprioceptive circuits with a significant delay. So, the neural circuits must be able to create a virtual compound ‘instant’ by extrapolating available sensory information (delayed) and anticipating movement, i.e., to build a ‘model’ of the ‘now’ instant (Fesce et al., 2024) that makes it capable of interfering with external objects — with external reality — in real time.

In the following, the main mechanisms through which a neuronal system can handle time intervals and time sequences will be discussed, together with the use the animal can make of these strategies to produce a consistent model of the external reality. In doing so it is necessary to compensate for processing delays, putting in register sensory elaboration with motor programming. A mathematical ***model*** of reality must be concocted, based on extrapolation, rather than accurate knowledge and representation. This modelling activity will be shown to give rise to the emergence of computational constructs that are related to the perception of identity and agency, which may well constitute the embryo of consciousness but are not a product of the latter.

## 3 Neuronal computation and time

The computational activity of neurons is based on the spatial and temporal summation of synaptic inputs onto their Soma and dendrites, to appropriately respond to specific patterns of synaptic activation. In order for a network to be able to identify a specific pattern in the incoming data stream, each neuron must be able to accurately select and isolate inputs that arrive precisely simultaneously. Since the time course of an excitatory post-synaptic potential (EPSP) typically spans a few ms (something more for PSPs generated by NMDA receptors or G-protein coupled receptors), synaptic events that are not exactly synchronous may sum up, possibly impairing the precise, discriminative perception of specific patterns of synaptic input convergence. Most inputs from the periphery reach the cortex through thalamic relay neurons; to overcome the problem of synchronicity, one mechanism exploits GABAergic (mostly parvalbumin-positive, PV) inhibitory interneurons. These neurons are associated to the principal (pyramidal) excitatory cells (PC) in the cortex, receive the same projections from thalamic neurons (TC), and produce an inhibitory PSP on the principal cell, which follows the EPSP by just about 1 ms (the typical delay introduced by one synapse). This way, the depolarization is rapidly shut off, and only synaptic activations that are truly synchronous—i.e., occurring within a ∼1 ms time window—can be summed to recognize a pattern (Tremblay et al., 2016). More precisely, distinct synaptic signals may be summed also if they arrive, separated by very short intervals, on synapses located at different distances from the cell body, so that they converge at the same moment on the axon hillock.

**FIGURE 1:**
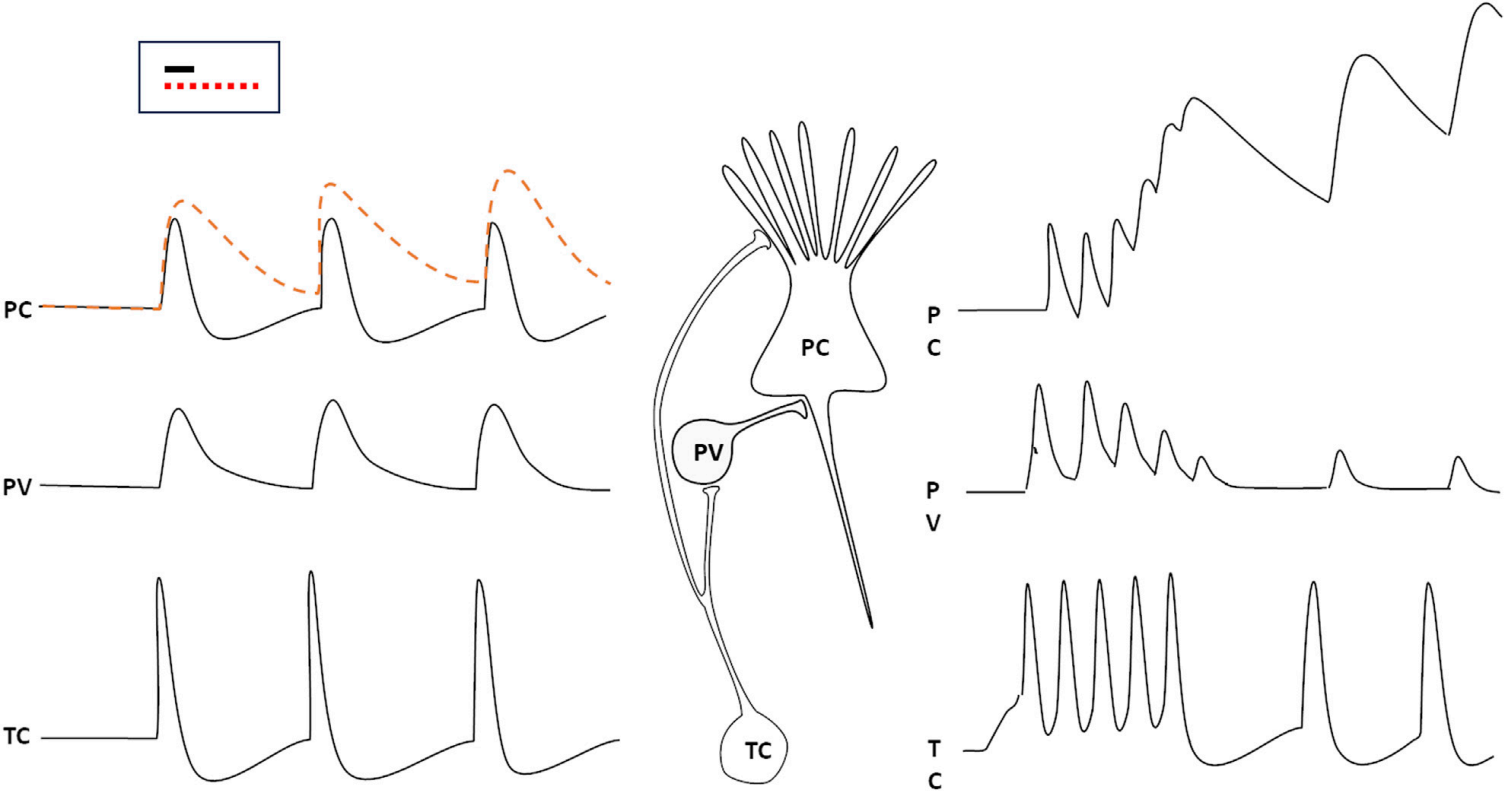
The responses of a parvalbumin positive GABAergic neuron (PV) and a cortical pyramidal cell (PC) to spikes generated by thalamic relay cells (TC). The PC would respond with a prolonged (several ms) EPSP to the stimulus (dashed red line) if the PV did not generate an IPSP just 1 ms (the typical delay introduced by a synapse) after the arrival of the thalamic input (black, continuous line). If the thalamic neuron were to discharge repetitively, the PV response would rapidly wane, so that the PC would add up impulses that arrive (at the same synapse or at neighbouring ones ones) at several ms intervals from each other (right). The inset displays the time window duration for synaptic input summation with properly functioning inhibition (black line) or when inhibition is impaired (red, dotted line).

If this circuit is impaired, as it happens when the thalamic neuron fires repetitively and the PV neuron adapts and fatigues, the time window for synaptic summation of the pyramidal cell lengthens, and the discriminative capability of the cortical circuit is lost (Figure 1, right hand side): this happens during slow wave sleep, but also in cortical circuits that are momentarily excluded from selective attention.

It might be observed here that the efficiency of this mechanism (narrowing the time window for signal summation), in addition of making cortical processing strictly discriminative, also reduces the spread of excitation, and this may bring the average branching parameter σ (number of other neurons activated by each neuron) close to the critical value of σ=1 , a condition of optimal information transmission (Beggs and Plenz, 2003), which also correlates with the presence of consciousness (Toker et al., 2022).

Detecting a pattern is not limited to extracting it from a set of data that arrive simultaneously: a pattern may as well consist in a precise sequence. If a sequence of signals are fed through distinct paths that introduce different, appropriate delays, they may be conveyed onto the same neuron so that they produce EPSPs that coincide precisely in time, and trigger the firing of an action potential; conversely, any other sequence in their occurrence would not generate such summation and would not be able to produce the firing by the target neuron.

**FIGURE 2:**
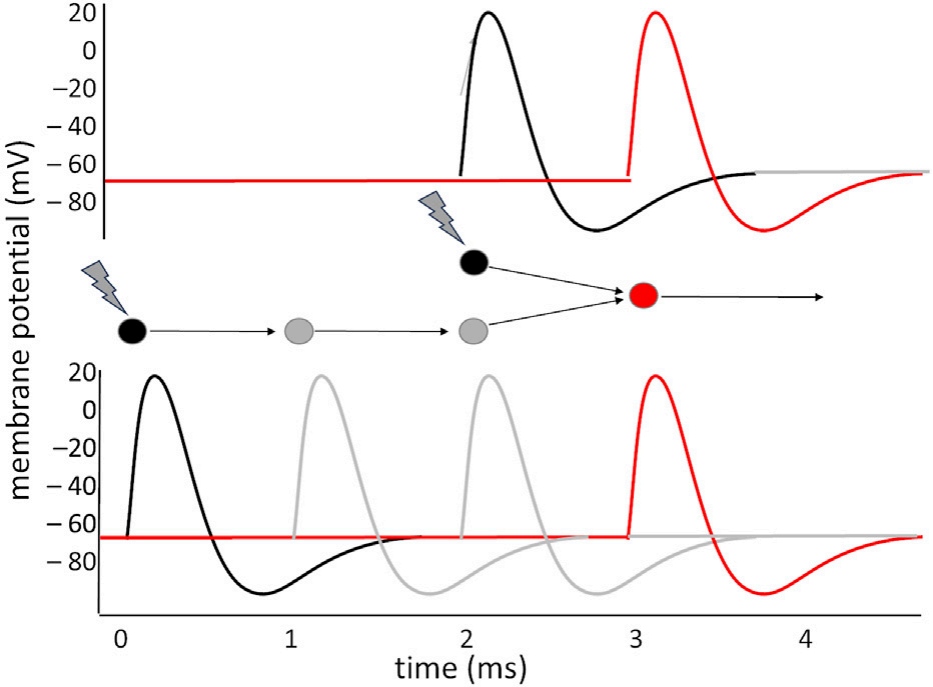
Making two asynchronous signals converge simultaneously on a neuron: two stimuli hit the black neurons with a 2 ms interval between them. The lower neuron receives the stimulus 2 ms before the other, but the two interposed neurons, in the lower circuit, introduce a delay of about 2 ms, which makes it so that the two paths converge onto the red neuron simultaneously, producing its activation. If the interval between the stimuli had been shorter, or longer, the red neuron would have not received the simultaneous convergence of two stimuli.

In general, the capacity of the neuron to isolate precisely synchronous inputs makes it possible to devise simple and complex time-interval — or frequency — detectors. Important applications of this principle are found in the visual system: direction sensitive (DS) ganglion cells in the retina exploit the Hassenstein and Reichardt (1956) detector model to determine the movement of an object in the visual field: information from two points in the retina are fed to two symmetric circuits, each of which introduces a delay in one of the two signals before combining it with the other onto the output neuron; if the object moves, the two signals will converge simultaneously in one circuit, while in the other they will arrive out of phase (Figure 2): the sign of the difference in the activation of the two output neurons indicates the direction of movement of the object. A similar principle is exploited in the visual areas of the cortex: the occipito-parietal area that elaborates movement (V5) compares visual information that arrives through the primary visual area (V1), and a series of processing steps in V2, V3 and V4 that delay it by some tens of ms, with the less precise but more direct visual information that arrives bypassing these elaboration stages, with a shorter delay.

## 4 Handling time

Handling time sequences is at least as important for motor control as it is for sensory elaboration. The cerebellum, in particular, has extraordinary capabilities in detecting coincidence; in limiting the time window for signal summation; and in precisely defining the timing for the activation of single muscles, when the complex interplay of the activations of agonist and antagonist muscles needs to be coordinated to produce a movement that starts smoothly, proceeds neatly, decelerates correctly, and stops at the target, without over-reaching (Spencer and Ivry, 2013). These properties — precise timing — and the presence of particularly numerous and plastic synapses on the main cerebellar cells (Purkinje neurons), sustain the sophisticated role of the cerebellum in movement monitoring and correction, in conditioning, in associative learning, and in transforming common, repeated, activities — motor as well as cognitive — into ‘automatic sequences’, that can be performed with high efficiency, speed and precision, and no need for attentive control.

The ability of precisely handling time intervals by the cerebellum can be exploited to produce delayed conditioned responses: it is possible to induce conditioned responses that arise after a precise time delay, up to several hundred ms, following the onset of the conditioned stimulus (delay or trace conditioning; Grover et al., 2022). This ability of the cerebellum indicates that the cerebellar circuit may also be able to produce predictions (see below: 7. Prediction in the temporal dynamics of physiological systems).

For longer time intervals, the mechanisms involved are not based on longer paths and synaptic delays. In most cases they exploit phenomena of synaptic facilitation and depression, which have decay times that range from tens of ms to tens of seconds. Examples can be found in what is called “sensory memory”: if the same sound is produced at regular interval, cortical responses gradually attenuate, as if the auditory cortex were keeping track of previously heard sounds and recognized the lack of any novelty; but a different sound tends to produce a larger cortical activation; this occurs for intervals between successive sounds up to 8–10 s (see, e.g., Tripathy and Öǧmen, 2018).

Timing and rhythms are not features of the nervous system only. In addition to the well-studied circadian variations (Healy et al., 2021), most systems in the body (e.g., cardiac and respiratory rhythms, muscular tone, besides EEG rhythms; Lehnertz et al., 2021) display continuous fluctuations in their activity. Correlations have been observed and analysed among the activities of these organ systems, pointing to the presence of functional links among them; the strength of such links can be quantified in various physiological states (e.g., wake vs. various sleep stages) by estimating the stability of the time-delay (TDS) in the correlations. The scale of these time delays ranges from several tens of seconds to less than one second; so these interactions—as well as the cardiac and respiratory rhythms—may contribute to timing neuronal activity and to perceiving time lapses. These findings have originated the new field of network physiology (Bashan et al., 2012) and shed a new light on the relations between organisms and time.

Time lapses longer than few second duration cannot be directly perceived and estimated by neuronal circuits: a certain level of abstraction is needed. Intrinsic pacemaker neurons and cerebellar circuits can produce precisely rhythmic activities and precisely timed repetitive behaviours (Spencer and Ivry, 2013). This helps in evaluating the duration of longer intervals (for example, we can estimate a 2-min interval by counting to 120).

In humans, since a large fraction of the brain is dedicated to vision and elaboration of visual patterns — objects and spatial localization — most cognitive aspects are handled by exploiting the computational capabilities of the parietal cortex, which is specialized in spatial computation and, consequently, geometrical and mathematical processing. Time itself is indeed handled in spatial terms, as a timeline along which events occur and develop. Humans also develop a cognitive construct that only a few animals seem to share, namely, object permanence. It is noteworthy that the idea that things (and people) exist and persist also when they are not present in our sensory sphere is not innate but develops as a cognitive construct by 1–2-year age (the exact time is controversial: see. e.g., Piaget, 1954; or Moore and Meltzoff, 2008).

Object permanence, combined with a pictorial representation of time as a line along which the events occur, makes it possible to handle time as a virtual space, to imagine its extension in the past and in the future, and to map on it the events we can recall as well as those that we are said have occurred. However, coherent with the fact that we cannot neurologically perceive long time intervals, there is no possibility of connecting the dots to reproduce the sequence of events of our life: we can recall many of them as isolated fragments of a movie, short flashes that we may or may not be able to position in the correct sequence along the timeline, in a virtual metaphoric space, depending on whether we can put them in a known temporal relation with relevant episodes that can be precisely located in time. This relation with time can be considered as a higher function, an aspect of consciousness, and the substrate of self-consciousness, because it constitutes the basis of our ability to recognize our own existence, extend the image of the self in time, and tell a story about reality and ourselves. However, an indispensable and basic function must be present in neuronal circuits for this capability to develop; even in the absence of what we call consciousness—whatever we mean by this word—neuronal systems must be intrinsically designed to model, i.e., to force the available information into a consistent reading.

## 5 In search of consistency

It has been some 60 years since the idea of ‘fuzzy logic’ was first advanced by Lotfi Zadeh of the University of California at Berkeley (Zadeh, 1965). It rapidly became a popular idea, at least among scientists, because it reflected the way the human mind works: based on imprecise, non-numerical, non-clear-cut information, and on degrees of truth (likelihood) rather than 1/0, yes/no, true/false oppositions.

Milestone experiments on the monkey visual cortex identified neurons that fired in response to the presentation or movement of slanted lines or profiles (Hubel and Wiesel, 1959; Hubel and Wiesel, 1968; Orban et al., 1981; Emerson et al., 1992). Although each neuron would fire preferentially in response to a line with a certain slant angle, it would respond to similar angles as well, the firing frequency gradually declining as the angle departs from the preferred one. This is, in a sense, a tentative recognition of a pattern, a fuzzy identification based on the likelihood that the pattern be actually there. Similarly, the revolutionary “hand” neuron reported by Gross (Gross et al., 1972) in the cortex of the monkey would fire when the animal was presented with a hand; with the drawing of a hand, from several different perspectives; also, though less intensely, with sketches that were reminiscent of a hand (a glove, even a glove with no fingers); but not with images obtained by scrambling the elements that constitute a hand. Once again, a fuzzy, tentative recognition.

These data indicate that the neuronal circuits implied in these ‘recognitions’ are essentially able to evaluate (and signal with their firing) how closely the sensory data resemble some meaningful pattern/object. That is, fuzzy recognition: the higher the likelihood, the more intense the firing. Therefore, these circuits appear to ‘model’ (or propose models), rather than “reproduce” or “depict,” what is out there: to try and interpret by analogy, by fitting an *internal scheme* to the external reality.

The whole neuronal elaboration is based on likelihood: parallel processing, many circuits, each looking for specific patterns, each possibly proposing the presence of the pattern they are supposed to identify. Nothing ever certain, and sometime conflicting possible interpretations. Thus, the logic of neural circuits is intrinsically fuzzy; the winning interpretation is based on the criterium “the least possible number and severity of inconsistencies.” As long as inconsistencies are there, regions of the limbic system—the anterior cingulate cortex in particular—are activated and sustain further analysis (Botvinick et al., 2001; Ullsperger and von Cramon, 2004), because consciousness asks for unambiguous readings.

So, neural systems, starting from the simplest nervous systems, must work based on an attempt at modelling (extracting and retaining the consistent structure in the available information) before reacting and even—if the nervous system is sufficiently complex–before interpreting.

Going back to the problem of timing, we pointed out the necessity of putting in register sensory inputs that arrive with several tens ms delay (and become conscious after hundreds of ms) with motor programs that must be produced tens of ms in advance, and with motor commands that also precede the actual movement. The nervous circuits must be able to extrapolate information about the external reality, in particular about rapidly moving objects, to predict future locations, and to precisely anticipate motor commands; only if this elaboration succeeds, positive reinforcement is received about the possibility of correctly interacting with the object. The perception is quite blurred and conflicting (the object does not appear to be there yet, the motor command has already been given, no proprioceptive feedback has been received yet); still, the modelling function must be able to extract from all this the consistent picture that the object is there, now, and the movement is occurring now. In other words, the modelling function must be able to generate the consistent perception that something is occurring exactly now (whatever this means) in the actual reality. This capability by the brain was thoroughly investigated by Libet et al. (1979), with particular focus on conscious elaboration: although the awareness of a stimulus arises with some 500 ms delay, conscious activity seems to be able to refer it back to the time the first cortical response was elicited by the stimulus (10–30 ms delay, depending on the location on the body, i.e., the distance to the cortex).

Obviously, unconscious and learned reactions can be much faster, but even in that case tens of ms delay in perception, and lead in motor anticipation, must be taken into consideration in building the “now” instant. All this does not require consciousness, *per se*. What is referred to here as the ‘modelling function’ precedes consciousness, occurs even if the animal is an absolutely dull animal to which we would never concede the benefit of a consciousness, such as a frog, or a primordial reptile, which is however capable of capturing a flying fly by correctly modelling reality, the movement of the fly, and its own motor capability, in such a consistent way that it actually succeeds.

## 6 A thought experiment

Imagine having to write a program to drive a tennis ball machine, which in addition to pitching must also be able to catch the ball hit back by the player. The program will have to include an algorithm to record perceptual information (P-algorithm); with a reasonable several-gigaHertz CPU, there is no practical delay in the depiction of reality. Still, a movement path must be designed to get the machine there in time to catch the ball, and this must be done sufficiently in advance to take into account delays due to the inertia of the machine; this also requires that the P-algorithm includes some predictive capability. Even if a precise action computation (A-algorithm) is performed, some impediments might arise (or a small error, or some unpredicted or hidden factor might have not been considered). It would be good to check the correctness of the predictions as well as the accuracy of the movement, on the fly. The prediction can be corrected, as can the motor program, but it is obvious that corrections in the P domain do not require actions while corrections in the A domain do. It is therefore clear that the two algorithms must behave differently, so that a computational construct must emerge in the elaboration, which differentiates the P-algorithm, that maps onto a “R = reality” domain that cannot be controlled by the program, from the A-algorithm, that maps onto a “I = identity” domain on which it does have control. So, a “P = perceptual” domain and an “A = agency” domain, as well as an “R = reality” domain and an “I = identity” domain emerge in the model with no need for any awareness. It may be noted that if the machine were also to take note of what happens, nobody would claim that the machine is able to consciously experience what is happening; still, something equivalent to the cognitive component of consciousness would take place — producing a representation of what is happening — and such representation would necessarily involve a distinction between the “P” and “A” algorithms, and between the “R” and “I” domains.

The thought experiment could be made slightly more complex if instead of catching a ball the problem were to intercept an asteroid in space, say 3,000 km away: the task would be similar, but a delay would also be present in the P domain (about 10 ms for the path the light has to travel). This would reproduce what happens in a nervous system, which receives sensory information with a macroscopically relevant delay (20 ms correspond to an error >10 cm for an object that travels at 20 km/h). In this framework, both the depiction of reality and the programming of actions require that delays be considered, but in this case as well the two domains must be treated differentially.

## 7 Prediction in the temporal dynamics of physiological systems

Most networks in physiological systems display time-dependent variations in their activity. In many cases, these constitute responses to changes in the external reality; however, quite often time-varying regulations are anticipatory, either based on predictable periodic fluctuations (seasonal or circadian oscillations) or on sensory inputs that suggest some later event (e.g., activation of gastric motility and secretion, and insulin release, upon simply seeing or smelling food). Thus, the capabilities of predicting and anticipating changes is not a privilege of the nervous system. Still, while most of the physiological networks in the organism face and produce changes in the time-scale of seconds, or longer, and the delays in the responses do not require that the temporal dynamics of internal or external processes be treated differently, the nervous system must deal with sub-second time-scales.

Rapid temporal dynamic handling by the nervous system strongly relies on the cerebellar circuits (and in particular the cerebro-cerebellum), which have been shown to be able to produce forward prediction: the output of the dentate nucleus cells predicts quite well the future input (through mossy fibres) that arrives 20 ms later, and reasonably well up to 100 ms (Tanaka et al., 2019). This is a strong indication that the cerebellum can overcome the problems raised by the sensory delay to the cortex; it can also generate preparatory activity lasting for several hundred milliseconds, which may regulate neuronal activity in the cerebral cortex to adjusts movement timing (Tanaka et al., 2020). However, it should be noted that the cerebro-cerebellum is the phylogenetically newest expansion in the cerebellum, and perfect synchronization of sensory-motor reactions also occurs in reptiles, which have a very rudimentary cerebellar expansion. It is generally thought that the optic tectum in reptiles (the superior colliculus of the midbrain in mammals) provides a mapping of external stimuli (visual and somaesthetic) and directly controls the rapid movements of the eyes and the head toward them; in mammals, this also elicits an arousal response and directs the attention to the stimulus. Graziano (2022) has equated this reaction to a primordial form of consciousness, proposing that consciousness consists in the capability of the brain to control attention and build a model of its own attentive activity (an “Attention Schema”); the fact that such model is imperfect would give rise to the fictitious perception that some metaphysical event is accompanying awareness. This would also account for the existence of subliminal stimuli, which would capture our attention but escape the registration by the imperfect model built by the Attention Schema/consciousness (Graziani, ibid.). Whether or not a model of the attentional activity—an Attention Schema—is built, and therefore whether or not any form of consciousness (à la Graziano) is present, a model of the external reality, and a distinct model of one’s own body and one’s controllable behaviour, must be there.

Animals that have a limited variety of motor behaviours may not need the sophisticated prediction capability displayed by the mammalian cerebellum, which may become more and more important when a wide assortment of motor schemes, and fine and precise movements, appear. As the cerebellum develops its capability of correcting movements on the fly, the difference between predicting the behaviour of an external object and designing, predicting, and correcting a motor behaviour becomes even more evident (and one should note that there is no awareness whatsoever of what happens in the cerebellum). The problem is not anymore a simple prediction problem: a backward extrapolation must be performed to design the appropriate movement trajectory and to translate it into an exact sequence of muscle activations (timing and intensity at any moment); then, the results of the movement must be compared with the program, and muscle activation may have to be modified to produce appropriate correction; this cannot be done very rapidly, because the proprioceptive and visual confirmation of the movement arrives with tens of ms of delay. The wonderful ability of the cerebellum consists in learning the procedure, and becoming able to finely tune it on the fly, even before a proprioceptive feedback returns, while in the same time reassuring the cerebral cortex that the movement is proceeding correctly (Spencer and Ivry, 2013). This avoids that intentional motor programming interferes, interrupting the fast and precise flow of the automatic execution of the learned movement. It should be clear how different this activity is from simply predicting and monitoring the trajectory of an external object, on which the cerebellum has no power. This is a further component of how the indispensable computational construct of “identity” must be there well before any consciousness arises of what is going on.

## 8 The emergence of identity and agency

The capability of modelling is an intrinsic skill of nervous systems; it helps make sense of the external reality, by identifying objects, examining their mutual relations, and conjecturing their possible relevance. In addition to this, computational networks must be able to extract fundamental coherent rules about what happens out there, so that future positions of moving objects and the consequences of motor commands can be extrapolated.

Identity, as the capability of differentiating what is self from what is not, is based on two properties of living organisms, the richness of sensory receptors in the body of any animal, and “affectability.”

Even unicellular organisms have receptors on the surface of their membrane; so, there is an objective distinction between anything that stays or occurs outside of the organism, and what gets in contact with it. The “self” is identically and inevitably distinct from the external reality, but sensory systems produce internal changes as a consequence of external ones, and an analytical system is needed to realize this distinction (and to conceptualize it): neural circuits must be intrinsically able to differentially handle information about external events or one’s own movement, but for this to become a mental construct a sufficiently complex neuronal network and sufficient training are required, as indicated by the fact that even a human new-born cannot appropriately distinguish their own body parts from external objects, until the baby effectively learns this, because the body generates sensations.

By “affectability” I indicate an intrinsic feature of every living system, namely, the presence of biophysical and biochemical mechanisms that produce a response when something relevant to survival, to the maintenance of homeostasis, to wellbeing, occurs and is sensed. If the organism is endowed with a nervous system, any sensation that has this kind of relevance will produce a change in the mode of operation of the nervous system: an increase in vigilance, arousal, alertness, reactivity, to a variable extent; responses might be accelerated; freezing, escape, fight, or consummatory, aggressive, predatory behaviours might be elicited. Whether we wish to call these changes in the mode of operation of the nervous system “pain,” “fear,” “rage,” “pleasure,” or we prefer to preserve these words to indicate the emotions of an animal with a sufficiently developed brain, their existence should be acknowledged; Damasio proposed that we consider them as the biological component of emotions—the “body marker”—as opposed to the cerebral, and conscious, elaboration, the “feeling” (Damasio, 1996).

To account for sensations and affectability, an implicit model must be built of the interaction of the self with the external objects: it ought to be the most consistent model of what is happening, and it is verified ex post, based on the result of behaviour. Success or failure will teach the computational modules, by producing plastic changes in the involved neurons and synapses. In building such model, two computational domains are generated: a perceptual (P) domain, which accounts for the external reality, and is affected by a variable delay (lag); and a motor control, action (A), domain, which handles behaviour, and must operate in advance by a variable time-lapse (lead).

The perceptual (P) domain is characterized by the possible interaction among sensory modalities: if an animal can see its own body, visual and somatosensory concurrence will signal that something particular occurs when a part of the body of the animal encounters an external object, and something different occurs when it encounters another part of its own body. This suggests that there exists a part of the world that has properties distinct from all the rest, because it gives rise to sensation; so, being able to model reality, even in the roughest mode, requires a self vs. non-self distinction. One may still claim that in the absence of consciousness such sophisticated modelling is not needed and is not there. A much stronger need for a distinction between self and non-self arises from the different computational requirements to deal with the temporal dynamics of the two domains mentioned above: whatever pertains to the former domain (P), and is not static, needs to be processed so to extrapolate from past information what may be the situation at this precise moment, whereas the elaboration in the latter domain (A) must take into account that the motor command have to be given in advance; also, whatever is elaborated in the (A) domain will produce a reverberation in the (P) domain after an appropriate time delay: perceiving the movement through proprioception and seeing the action that is being performed and produces its consequences.

Whether a brain has a ‘consciousness’ that can tell it, or not, a time lag and a difference in the possibility of control separates the two domains of neuronal activity and has to be taken into consideration to model, and appropriately interact with, the external world. This constitutes a computational and logical basis of “identity,” which therefore emerges as a *computational construct*, necessary to interact with the external reality in real time. This construct must permeate the activity of any brain, even a bee’s brain, whether awareness of such “identity” is there or not.

Agency, as the capability of realizing that one is the subject of their own sensations, emotions and thoughts, and the agent of their own acts, is a much more complex function, but it must be present in some rudimentary, implicit form, for any animal that is able to elaborate a behavioural strategy, in hunting, fighting and generally in interacting with other animals or objects. In fact, a further crucial consequence of the abovementioned computational duality consists in the fact that the motor commands produce perceivable consequences. Several neurotransmitters and mechanisms of potentiation or depression of synaptic contacts are associated with success or failure of the action; this results in either positive or negative reinforcement, i.e., plastic changes in the neuronal networks that account for the capability of modifying behaviour, based on success or failure of previous actions; this leads to the accumulation of heuristics that will guide behaviour in a successful way. Computationally, this requires that an implicit causal connection be assumed between behaviour and subsequent events/sensations. Combined with the need for the computational construct of “identity,” the detection of a causal connection between motor control (A) and consequent changes in the surrounding reality (P) generates another *computational construct*: “agency,” as a causal relation between the temporally separated domains of motor control and perception.

So, neuronal networks display the capability of isolating precisely synchronous signals, handling time delays and time sequences, in the ms-to-second range, and modelling interaction with the external world, by taking into account delays in sensory elaboration and motor programming.

The modelling activity requires that two computational domains (perception vs. motor control) be dealt with on two distinct time scales. This, combined with somatosensory information, and in particular with the proprioception that follows movement, gives rise to the emergence of ‘identity’ as a computational construct. The heuristic selection of behaviours based on reinforcement and neuronal plasticity establishes causal relations between motor control and the consequence of actions. This gives rise in a similar way to the emergence of ‘agency’ as a computational construct.

## 9 What about consciousness, then?

All this comes about with no need for any form of awareness.

If a brain is capable to build a sufficiently accurate model of reality and the self, these computational constructs — identity and agency — evolve into criteria to interpret reality, events, and one’s own sensations, emotions, actions, thereby significantly contributing to the emergence of consciousness and self-consciousness.

In the scientific arena we should be careful in using the term “consciousness” and in attributing functions to it. The activation of sensory systems, and the capability of recognizing and attributing a meaning to the activation of a sensor, are functions that a machine can easily perform and do not imply awareness; so, the words “sensing” and “perceiving” should not be directly associated with the idea of “consciousness” (Fesce, 2023). Alertness and responsiveness are sometime considered as synonyms of consciousness, but a fly displays them when it escapes your hand that tries to smash it; they do not require awareness; they may be required for consciousness to be there, but are not brough about by consciousness. The capability of producing a representation of reality may also be an aspect of consciousness, but it is a mere cognitive (informational) function that can be achieved by an AI system, and even by any computer, and therefore is not a specific property of consciousness. The generation of a subjective perspective in the analysis of reality characterizes the elaboration by the sensory-motor areas of the cortex and is therefore an intrinsic feature of the nervous system (Fesce, 2020) that precedes the onset of awareness: it is not a specific feature of consciousness. Here we have discussed how identity and agency emerge as necessary computational constructs in processing and modelling by the nervous system of a moving organism, in order to reconcile the delay in perception with the anticipation (and control) needed for motor programming; possibly, this was already very obvious for a neurobiologist, but it contradicts the common sense that identity and agency are specific properties of consciousness (or even define it).

So, sensing, perceiving, recognizing, interpreting (giving meaning), modelling, as well as subjectivity, identity and agency, are all intrinsic functions or emergent properties of neuronal computation, which are present with no need for consciousness or awareness. In defining human consciousness, we consider further aspects: alertness, responsiveness, sharpness and soundness of thought, orientation in space and time, exam of reality, consistency between thought and emotions. However, these are just qualitative or quantitative features that allow us to evaluate the presence, intensity, effectiveness of consciousness, and whether it operates in a correct or defective way. They do not constitute consciousness.

If consciousness is stripped of these collateral aspects, it reduces to two clear specific functions.

The first is an affectability function (a property of the body of any living organism rather than of the brain or mind), that consists in the organism inevitably being *affected* by any experience (or internal activity) that has some relevance to its wellbeing (Fesce, 2023). This produces biological changes (functional, adaptive or preparatory, and communicative) that Damasio (1996) defined as the “body marker” of emotions, but in the meantime changes the operating mode of the nervous system: arousal, responsiveness, and possibly, in sufficiently complex brains, the onset and cognitive elaboration of feelings and possible rearrangement of motivational drives in the control of behaviour.

The second would be a merely cognitive function, consisting in the attempt at producing a full model, as fitting and consistent as possible, of reality; if a sufficiently refined nervous system is there, the model will consider identity and agency as well, will include the self, possibly consciousness itself, and in particular the just mentioned capacity of being affected. This is essentially what Graziano (2022) refers to as the “attention schema,” i.e., the specific aspect of neural modelling that controls attention and produces a model of such control process; Graziano claims that due to its being imperfect—as all neural models are—the schema would represent itself as the product of an internal metaphysical activity, and this way he dismisses the so-called “hard problem of consciousness”—why and how do we transform sensations into subjective, personal and private experiences—as a non-problem. Still, the capacity of being affected is there, far from being an illusion, a ghost generated by a misinterpretation by the attention schema.

Given the obvious connections between the cognitive aspects of consciousness and the mechanisms of selective attention, most neuroscientists see awareness as the result of a mental process being able to invade some areas of the brain (be they mostly anterior-frontal rather than posterior-parietal areas, according to views of the prevailing schools) and become able to resonate in the global neuronal workspace (global workspace theory, GWT; see, e.g., Dehaene et al., 2014). Information that gains the focus of selective attention is elaborated in a discriminative way, thanks to the activity of inhibitory interneurons that narrow the time window for signal summation in the involved cortical circuits (Tremblay et al., 2016); this may produce the degree of local inhibition that is appropriate to bring the network branching parameter close to the critical value σ=1 (Beggs and Plenz, 2003), so that such information may be effectively transmitted, and thus invade other areas of the brain and give rise to consciousness (Toker et al., 2022).

Several systems display features that have been associated with the idea of consciousness: artificial intelligence systems are able to produce an internal model of reality and enact complex regulations to adequately interact with it; a bee colony is able to share the representation of the surroundings through the dances of the bees getting back from an exploration; the ant community that constitutes an anthill is capable of incredibly complex finalized behaviours; organisms, in general, behave as network systems capable of performing complex developmental programs and adapt to the external reality. In relation with what has been discussed here, it might be observed that electronic systems are able to acquire data and react on a sub-millisecond time scale, so they almost never need to apply different computational treatments to the temporal dynamics of external events or of their own elaboration and responses. Vice versa, bee colonies and anthills do not need to react (as a complex unit/organism) in real time with rapidly changing external events, and adaptations of the system networks in the organism generally occur on the same time-scale as external changes. So, in none of these cases the computational constructs of identity and agency are needed to handle differential temporal dynamics for correct performance. This suggests that not only identity and agency might be there before consciousness, but also some system networks can appear to behave in a conscious way though they do not even consider identity and agency.

## 10 Conclusion

By ‘consciousness’ we may refer to a state — being conscious rather than absent — or to a property that some animals possess (possibly only some humans), or to a topological portion of the mind, more or less clearly recognizable from its pre-, sub-, un-conscious domains, or finally to a function, which is able to transform a sensation into a personal experience.

Whatever we mean by that term, even in the absence of all these aspects a nervous system must be able to model the reality in a way sufficiently consistent to survive. To that end, time delays that are implicit in sensory processing and motor programming must be considered in modelling reality. Thus, the *modelling function* is bound to signal the specificity of the body with respect to the rest of reality (identity) and the ownership of the actions and sensations (agency).

In conclusion, identity and agency are not byproducts of awareness, but constitute intrinsic necessities, in the form of cognitive constructs, of the modelling function by the nervous system. They are emergent properties that may evolve into mental (conscious) constructs in a brain capable of awareness. The capacity of transforming a sensory or cognitive activity into a personal, private experience—what is referred to as the “hard problem of consciousness”—builds on the emergent features of neuronal network processing that precede and characterize consciousness: sensation, perception, alertness, responsiveness, subjectivity, identity and agency. Consciousness emerges when all these properties are coloured by emotional elaboration and encounter the continuous endogenous production of imaginative activity (Fesce, 2023).

## Data Availability

The original contributions presented in the study are included in the article/supplementary material, further inquiries can be directed to the corresponding author.
